# Interventions for increasing ankle joint dorsiflexion: a systematic review and meta-analysis

**DOI:** 10.1186/1757-1146-6-S1-O41

**Published:** 2013-05-31

**Authors:** Rebekah Young, Sheree Nix, Aaron Wholohan, Rachael Bradhurst, Lloyd Reed

**Affiliations:** 1 School of Clinical Sciences, Queensland University of Technology, Brisbane, Australia; 2 Foot Fitness Podiatry Clinic, Brisbane, Australia; 3 Institute for Health and Biomedical Innovation, Queensland University of Technology, Brisbane, Australia

## Background

Ankle joint equinus, or restricted dorsiflexion range of motion, has been linked to a range of pathologies of relevance to sports medicine practitioners. This systematic review and meta-analysis investigated the effects of conservative interventions on ankle joint range of motion in healthy individuals and athletic populations.

## Methods

Keyword searches of Embase, Medline, Cochrane and CINAHL databases were performed. Studies were eligible for inclusion if they assessed the effect of a conservative intervention on ankle joint dorsiflexion in healthy populations. Papers were quality rated using a standard quality assessment scale. Standardised mean differences (SMDs) and 95% confidence intervals (CIs) were calculated and results were pooled where study methods were homogenous.

## Results

Twenty-three papers met eligibility criteria, with a total of 717 study participants. Results suggest that there is some evidence to support the efficacy of static stretching alone (SMDs: range 0.70 to 1.69) and static stretching in combination with ultrasound (SMDs: range 0.91 to 0.95), diathermy (SMD 1.12), diathermy and ice (SMD 1.16), heel lifts (SMDs: range 0.7 to 0.77), superficial moist heat (SMDs: range 0.65 to 0.84) and warm up (SMD 0.87) in improving ankle joint dorsiflexion range of motion.

## Conclusion

Some evidence exists to suggest the efficacy of stretching programs as well as the combined use of stretching and ultrasound, diathermy, diathermy and ice, superficial moist heat, warm up and heel lifts in increasing ankle joint range of motion. These interventions may be beneficial in preventing or managing pathology in individuals with restricted ankle range of motion. There is currently a paucity of quality evidence to support the efficacy of other conservative interventions.

